# Unraveling the synergy: how organizational intelligence fuel soft skills and nurses’ thriving: a cross-sectional study

**DOI:** 10.1186/s12912-024-01933-w

**Published:** 2024-05-08

**Authors:** Amal Diab Ghanem Atalla, Naglaa Abdelaziz Mahmoud Elseesy, Ayman Mohamed El-Ashry, Loujain Saud Sharif, Alaa Mahsoon, Wafaa Farraj Aljohani, Samia Mohamed Sobhi Mohamed

**Affiliations:** 1 Department of Nursing Administration, Faculty of Nursing, Alexandria University, Alexandria, Egypt; 2 Department of Public Health Nursing, Faculty of Nursing, King Abdulaziz University, Jeddah, Saudi Arabia; 3 Department of Psychiatrc And Mental Health Nursing, Faculty of Nursing, Alexandria University, Alexandria, Egypt; 4 Department of Psychiatric and Mental Health Nursing, Faculty of Nursing, King Abdulaziz University, Jeddah, Saudi Arabia; 5 Department of Medical Surgical Nursing, Faculty of Nursing, King Abdulaziz University, Jeddah, Saudi Arabia; 6 Nursing Program, Batterji Medical College, Jeddah, Saudi Arabia

**Keywords:** Organizational Intelligence, Soft skills, Thriving at work, Nurses

## Abstract

**Aim:**

Investigate the influence of organizational intelligence on the development of soft skills and the overall thriving of nurses at Alexandria Main University Hospital in Egypt.

**Design:**

A cross-sectional descriptive design following STROBE guidelines examined the relationship between organizational intelligence, soft skills, and nurses’ thriving.

**Methods and tools:**

Data were collected from 740 nurses working across critical care units using structured questionnaires. The questionnaires assessed organizational intelligence, soft skills, and thriving at work. Sociodemographic characteristics, including age, gender, education, and nursing experience, were also collected. Statistical analyses were used to analyze the data, including ANOVA, t-tests, Pearson correlation, and stepwise regression.

**Results:**

The findings revealed nurses’ positive perceptions of organizational culture and documentation skills. However, areas such as measures and rewards and staff relations and communication indicated opportunities for improvement. Nurses reported high vitality levels but needed more learning opportunities at work. Significant correlations were found between demographic variables, organizational intelligence, soft skills, and thriving. Organizational intelligence demonstrated a robust positive relationship with both soft skills and thriving. Age, gender, education, and experience significantly influenced nurses’ soft skills and thriving.

**Conclusion:**

The study highlights the importance of organizational intelligence in enhancing nurses’ professional capabilities and well-being. Addressing demographic factors and fostering a supportive work environment is crucial for optimizing nursing practice and organizational effectiveness.

**Nursing implications:**

Insights from this study can inform targeted interventions and policy decisions to enhance nursing practice, organizational development, and healthcare outcomes in Egypt. Fostering organizational intelligence and soft skills among nurses can improve patient care, increase job satisfaction, and overall organizational success.

## Introduction

The conditions of working life are determined by a multitude of variables lately. While technological advancements, globalization, shifts in the expectations of individuals, unanticipated upheaval, and crises demand change and creativity, they additionally render managing working conditions more difficult. The healthcare organization and its employees’ ability to embrace innovation and be risk-averse is correlated with their high organizational intelligence, openness to new ideas, and readiness to take on new ones [1, 2]. Effectively managing and coordinating information and ideas to satisfy patients’ needs is known as organizational intelligence. By combining technical and human resources, an organization can solve organizational challenges through intellectual talent [3, 4].

Organizational intelligence (OI) is one of the most critical capabilities of a healthcare organization, and it increases its changeability. According to McBreen et al. (2022, p. 125 − 33), “the core capability of an organization in making wise choices and intelligent decisions based on available knowledge and analytical power; it is an organization’s ability to innovate, create, and remain productive and competitive [5]. Additionally, OI is considered necessary for healthcare organizations to succeed and aids in their purpose accomplishment [6, 7].

Effective organizational intelligence aids in highlighting the positive aspects of an organization while highlighting its weaknesses. Rather than assessing each member of a group’s intelligence, organizational intelligence articulates the idea of intelligence for the entire organization [1, 8]. This study adapted Falletta’s OI model (2018) to assess OI perception. In the model, the variables situated in the upper section, such as environmental inputs, have an external impact on the healthcare organization. The three strategic drivers of the healthcare organization are leadership, strategy, and culture. Employee engagement and performance outputs are directly influenced by the healthcare organizational capability variables, which are the main drivers of employee engagement and healthcare organizational effectiveness [8].

In healthcare organizational intelligence, nurses are expected to possess hard and soft skills competencies as professionals. All employees, especially nurses, should have soft skills [9]. It is imperative to acknowledge that success is contingent not only on intellectual abilities but also on possessing soft skills critical to achieving professional goals [10]. Soft skills refer to the combination of personal traits and social skills that an individual possesses, which make them a desirable service provider. These skills are crucial for personal growth, social participation, and professional success. In today’s job market, non-technical or soft skills are becoming increasingly important. These skills include self-improvement, effective communication, and transferable abilities that are human-focused and interactive [11]. Thus, soft skills strengthen the ability to control personal emotions, flexibility to confront changes and adapt, optimism, invention, and initiative, which makes it simpler for nurses to perform their nursing jobs [12]. Today, healthcare organizations must consider the soft skills of nurses because nurses are more sensitive and affected by their work environment, so they can experience thriving and flourishing [13].

One definition of “thriving” in nursing is “place-related well-being” about the individual and the larger institutional, relational, and environmental context. Experiencing ill-being (i.e., limitations in one’s physical, functional, or cognitive abilities) does not always prevent one from thriving because one can simultaneously perceive one’s life as positive. This is because the definition of thriving is mainly subjective [14].

Thriving presented a two-dimensional definition of thriving consisting of (vitality), a sense of life, and (learning) the perception that one is improving or learning new things. Being energetic and having a passion for one’s work is known as vitality. The learning dimension denotes gaining and using information and abilities to increase capacity and self-assurance. When combined, The two dimensions—affective (vitality) and cognitive (learning)—combine the fundamental components of the psychological experience of personal growth [15].

The fundamental premise of flourishing at work is employees’ high levels of vitality and learning needs. Vitality and learning can suggest specific improvements to attain growing and own progress at work; however, they complement each other to create a thriving experience. Work thriving is negatively correlated with intentions to leave an employer and positively correlated with commitment, job satisfaction, self-development, and civic engagement. Thriving produces creative performance and favorable employee outcomes regarding development and health [13, 16, 17].

According to McBreen et al. (2022), organizational intelligence is a great way to increase soft skills and thriving among nurses and reduce staff turnover and leave intention. Corporate intelligence has gained much traction in human resource management as a dynamic method of enhancing work structures and procedures with an atmosphere that promotes nurses’ autonomy, flexibility, nurse development, and job thriving. The secret to keeping staff members happy, inspired, and focused on their achievements is to do this and their healthcare organization. Hence, this study aims to investigate the association between organizational intelligence and nurse soft skills, which enhances nursing thriving to become a healthcare organization more productive [5].

### Significance of the study

This study holds significant relevance for the nursing profession in Egypt. It addresses a notable and crucial gap in the existing literature by explicitly focusing on the relationship between organizational intelligence, soft skills, and nurses’ thriving in Egypt. This is significant as it provides insights and evidence-based knowledge tailored to the unique challenges and dynamics of the Egyptian healthcare system. By understanding the synergy between organizational intelligence and soft skills, healthcare organizations and policymakers can design targeted interventions and training programs to foster these attributes among nurses. This, in turn, can lead to improved patient care, increased job satisfaction, and enhanced overall performance in the nursing profession.

Instantaneously, the study on the synergy between organizational intelligence and soft skills in nurses’ thriving has excellent significance for Egypt’s nurses. Enhancing nurses’ skills and knowledge through continuous education and professional development programs can contribute to their thriving. Recommendations include promoting lifelong learning, providing opportunities for nurses’ skill-building, and offering mentorship or coaching programs. Creating a positive and supportive work environment is crucial for nurses’ well-being. Recommendations may involve fostering strong leadership, promoting teamwork and collaboration, improving communication channels, and implementing strategies to address work-related stress and burnout.

Ensuring a healthy work-life balance is essential for nurses’ well-being through implementing flexible scheduling options, promoting self-care practices, and providing resources for managing stress and maintaining a healthy lifestyle. Also, recognizing and appreciating nurses’ contributions can boost their motivation and job thriving through establishing reward and recognition programs, acknowledging achievements, and providing opportunities for career advancement. Empowering nurses’ voice: Encouraging nurses to have a voice in decision-making processes and engaging them in organizational changes can enhance their sense of ownership, soft skills, and job thriving, including promoting participatory decision-making, creating forums for feedback and suggestions, and fostering a culture of open communication.

Moreover, the study underscores the importance of organizational intelligence in creating supportive work environments and Promoting organizational development. By recognizing the significance of administrative support and leadership, healthcare institutions in Egypt can focus on optimizing their structures, policies, and practices to foster a culture that values continuous learning, collaboration, and personal growth. This can result in higher levels of nurse thriving and overall organizational success. The researchers hope that the research outcomes can inform policy decisions related to nursing education, professional development, and healthcare management in Egypt. Policymakers can use the study’s findings to develop guidelines and frameworks that prioritize integrating soft skills and organizational intelligence in nursing curricula and competency frameworks. This can lead to a more comprehensive and practical nurse training and development approach, ultimately benefiting the healthcare system.

### Aim of the study

- Investigate the influence of organizational intelligence on the development of soft skills and the thriving of nurses at Alexandria Main University Hospital in Egypt.

### The research question

- What is the relatioship between organizational intelligence, soft skills, and thriving of nurses at Alexandria Main University Hospital in Egypt?
- What is the influence of organizational intelligence on the development of soft skills and the thriving of nurses at Alexandria Main University Hospital in Egypt?

## Methods

### Study design

A cross-sectional descriptive design was adopted Following STROBE guidelines.

### Setting

The study was accomplished at Alexandria Main University Hospital. It provides free public health services and has more than 6,760 beds available. It is Alexandria’s largest educational university hospital. All 23 critical care units—the Second, Third, Fourth, Medical Emergency, Surgical Emergency, Intensive Care Unit of Emergency Operation, Transitional ICU, New Transitional Unit, Continuous Renal Replacement Therapy and Critical Cases ICU, Toxicity Unit, Maxillo Facial and Plastic Surgery ICU, Burn ICU, Pulmonology ICU, Neurosurgery ICU, Neurosurgery ICU, Neurosurgery ICU for Pediatrics, Hematemesis ICU, Urology ICU, Anesthesia ICU, Systemic Lupus ICU, Hepatic Transitional ICU, Diabetics ICU, as well as Ear, Nose, and, and Trachea (ENT ICU).

### Sampling

All target populations of nurses were included in the study; this is a purposive sampling strategy in which we decide to look at the complete population (i.e., the total population) that meets a specific set of requirements (inclusion criteria). Nurses who had worked in the units above for at least a year were included in the study (*n* = 740) as participants to increase their familiarity with the hospital system, administrative rules, policies, and regulations. The nurses also had to be present during the data collection period. Nurses who met the following criteria were chosen to participate in the study: (1) they had to be employed in previously chosen settings; (2) they had to have worked in the working unit for at least a year; and (3) they had to be directly providing patient care.

### Study tools

#### Sociodemographic characteristics section

The researchers asked questions about the study participants’ years of work unit, gender, age, education, and nursing experience.

#### Organizational intelligence scale

It was developed by Falletta and Combs (2018) to assess nurses’ perception of organizational intelligence [8]. It includes 52-item grouped into eleven main dimensions, namely; environmental inputs (2-items), leadership (5-items), strategy (5-items); culture (7-items), structure/decision rights (5-items), information/technology (4-items), direct manager (5-items); measures /rewards (7-items); growth/ development (3-items); staff nurses’ engagement (6-items); and performance outputs (3-items). Responses to the questionnaire were measured on a 5-point Likert rating scale extending from [1] strongly disagree to [5] strongly agree. The Organizational Intelligence Questionnaire (OIQ) score range varies from (52 to 260). The researchers tested the tool’s validity through an exploratory factor analysis. The loading score ranged from 0.512 to 0.966 before and 0.544 to 0.951 after varimax rotation. This accounted for 82.151% of the total variance, more significant than 0.35. The Kaiser-Meyer-Olkin measure of sampling adequacy was 0.923, indicating that the data was suitable for factor analysis. Additionally, Bartlett’s test of sphericity reached statistical significance (*P* = 0.000), which confirmed the factor ability of the correlation matrix. Therefore, the items of the scale were retained. In the present study, Cronbach’s alpha was 0.95.

### Career soft skill scale

This tool was created by Hussein Yassein & Samir Abd El-Aziz Elsaiad (2021) to evaluate nurses’ soft skills. It has 27 items and five dimensions: two for innovation, six for staff communication, seven for patient communication, seven for documentation, and five for staying technically current [18]. The responses were measured on a 5-point Likert scale, fluctuating from strongly agree [5] to disagree [1] strongly. The overall score ranges from 26 to 130. The researchers tested the tool’s validity through an exploratory factor analysis. The loading score ranged from 0.662 to 0.912 before and 0.598 to 0.982 after the varimax rotation. This accounted for 79.45% of the total variance, more significant than 0.45. The Kaiser-Meyer-Olkin measure of sampling adequacy was 0.923, indicating that the data was suitable for factor analysis.

Additionally, Bartlett’s test of sphericity reached statistical significance (*P* = 0.000), which confirmed the correlation matrix’s factor ability. Therefore, the scale items were retained. Cronbach alpha in the current study was 0.93.

### Thriving at work scale

This tool was developed by Porath et al. (2012) to assess nurses’ perception of thriving at work [15]. It consisted of 24 items, which consisted of two dimensions: vitality dimension (10 items) and learning dimension (14 items). The responses were measured on a 5-point Likert scale ranging from strongly agree [5] to disagree [1] strongly. The overall score ranges from 24 to 120. The researchers tested the tool’s validity through an exploratory factor analysis. The loading score ranged from 0.451 to 0.901 before and 0.501 to 0.902 after the varimax rotation. This accounted for 80.151% of the total variance, more significant than 0.35. The Kaiser-Meyer-Olkin measure of sampling adequacy was 0.903, indicating that the data was suitable for factor analysis. Additionally, Bartlett’s test of sphericity reached statistical significance (*P* = 0.000), which confirmed the factor ability of the correlation matrix. Therefore, the items of the scale were retained. In the current study, Cronbach’s alpha was 0.82.

### Ethical considerations

The study protocol was accepted by the faculty of nursing, Alexandria University Research Ethics Committee. Nurses remained informed about the drive of the study before providing their written consent. A code number was assigned to each questionnaire to ensure privacy and anonymity. The usage of data was restricted to research, as promised to nurses. The option to opt out of the study has been validated.

### Pilot study

10% of nurses (*n* = 74) approved the pilot study to identify potential obstacles and problems during data collection and to protect the goods’ practicality and simplicity. Nothing needed to be changed. Those who participated in the pilot study were left out of the study. The researchers examined the completed questionnaires for accuracy and inclusivity.

### Data collection

The participants received personalized copies of the study questionnaires. Each nurse received a hand-delivered questionnaire from the researchers, who then gathered the completed forms. Following a one-on-one, two-minute interview with each nurse to explain the purpose of the study, they were requested to give it back to the researcher. To guarantee the respondents’ objectivity, the purity of their opinions, and the completion of all questions, these scales were filled out in front of the researcher. Monitoring the delivery and gathering to ensure the highest response rate was simple because they were linked to specific working units. For their involvement, participants were given tiny snacks. The estimated time to fill out the questionnaires was 15–20 min. It took three months, from April 2023 to July 2023, to collect the data. All queries from the nurses were addressed, and clarifications were provided.

### Data analysis

The computer was fedded with the data, and IBM SPSS software package version 23.0 was used for analysis. A one-way ANOVA test was employed for comparisons between more than two categories. Two categories were compared using the student t-test for regularly distributed quantitative data. The Pearson coefficient was employed to correlate customarily distributed quantitative data. At the 5% level, the results’ significance was assessed. The internal consistency of the scale was calculated through Cronbach’s α coefficient.

## Results

Table 1 displays the sociodemographic and clinical details of the 740 nurses under study. Most nurses, 28.2% (209), were aged between 30 and 40. Of the total, 30.7% (227) were male and 69.3% (513) were female. In terms of marital status, 20.1% (149) were single, 63.5% (470) were married, 8.1% (60) were divorced, and 8.2% (61) were widowed. As far as qualifications are concerned, 40.7% (301) held a nursing diploma, 28.5% (211) were nursing technicians, and 30.8% (228) had a Bachelor of Nursing degree. Regarding years of experience as a nurse, approximately 30.1% (223) had 1 to less than 5 years of experience, followed by 28.1% (208) with 15 years or more of experience. Lastly, in terms of years of experience in the current hospital, 36.9% (273) had 1 to less than 5 years of experience, while 25.3% (187) had 15 years or more of experience.

**Table 1:** The socio-demographic and clinical data of the studied nurses (*n* = 740)

socio-demographic characteristics	No	%
**Age (years)**		
< 20	116	15.7
20–30	168	22.7
> 30–40	209	28.2
> 40–50	122	16.5
> 50	125	16.9
**Gender**		
Males	227	30.7
Females	513	69.3
**Marital status**		
Single	149	20.1
Married	470	63.5
Divorced	60	8.1
Widow	61	8.2
**Qualification**		
Nursing diploma	301	40.7
Nursing technicians	211	28.5
Bachelor of nursing	228	30.8
**Years of experience as a nurse (years)**		
< 1	21	2.8
1- <5	223	30.1
5 - <10	165	22.3
10 - <15	123	16.6
≥ 15	208	28.1
**Years of experience in this hospital**		
< 1	54	7.3
1- <5	273	36.9
5 - <10	124	16.8
10 - <15	102	13.8
≥ 15	187	25.3

Table 2 shows the mean score of Organizational Intelligence, soft skills, and thriving at work among nurses. In the Organizational Intelligence Scale, the Culture dimension scored the highest mean score (3.70 ± 0.67), indicating that this aspect was most strongly represented in the organization. However, the Measures & Rewards dimension scored the lowest mean score (3.18 ± 0.95), suggesting this area could be improved. In the Soft Skill Scale, nurses excelled in the Documentation dimension, which scored the highest mean score (4.07 ± 0.77). On the other hand, the Staff relations and communication dimension scored the lowest mean score (3.88 ± 0.76), indicating this area could be improved. In the Thriving at Work Scale, the Vitality dimension scored the highest mean score (3.71 ± 0.36), indicating that nurses felt energized and alive at work. However, the Learning dimension scored the lowest mean score (3.53 ± 0.43), suggesting there may have been room for improvement in promoting learning opportunities at work.

**Table 2:** Overall status of Organizational intelligence, soft skills, and thriving at work Scales among nurses (*n* = 740)

Variables	Total score	Mean score
**Tool I: Organizational intelligence Scale**		
Environmental Inputs	7.24 ± 1.73	3.62 ± 0.87
Leadership	17.72 ± 3.71	3.54 ± 0.74
Strategy	16.60 ± 4.0	3.32 ± 0.80
Culture	25.93 ± 4.68	3.70 ± 0.67
Structure & Decision Rights	17.91 ± 4.06	3.58 ± 0.81
Information & Technology	14.58 ± 3.03	3.64 ± 0.76
Direct Manager	17.78 ± 3.38	3.56 ± 0.68
Measures & Rewards	22.27 ± 6.64	3.18 ± 0.95
Growth & Development	9.96 ± 2.68	3.32 ± 0.89
Staff Engagement	20.21 ± 4.87	3.37 ± 0.81
Performance Outputs	10.39 ± 2.48	3.46 ± 0.83
**Overall Organizational intelligence questionnaire**	**180.57 ± 32.81**	**3.48 ± 0.63**
**Tool II: Soft skill Scale**		
Staff relations and communication	23.30 ± 4.55	3.88 ± 0.76
Communication with patient	27.53 ± 6.67	3.93 ± 0.95
Documentation	24.39 ± 4.60	4.07 ± 0.77
Innovation	7.92 ± 1.57	3.96 ± 0.79
Keeping up-to-date technically	19.24 ± 2.94	3.85 ± 0.59
**Overall Soft skill evaluation**	**102.38 ± 17.90**	**3.94 ± 0.68**
**Tool III: Thriving at work Scale**		
Vitality dimension	37.11 ± 3.65	3.71 ± 0.36
Learning dimension	49.41 ± 5.97	3.53 ± 0.43
**Overall Thriving at work**	**86.52 ± 8.47**	**3.62 ± 0.35**

*Note*: Data are *Mean* ± *SD*

Table 3 presented the findings regarding the correlation between sociodemographic varieties and Organizational Identification (OI), soft skills evaluation, and thriving at work among a sample of 740 participants. Participants under 20 had the lowest OI and soft skills scores, while those over 50 had the highest. Older age groups also scored higher in thriving at work. The differences were significant (F = 25.640, *p* < 0.001 for soft skills; F = 23.006, *p* < 0.001 for thriving at work). Females scored higher in soft skills and thriving at work than males (*p* < 0.001). Divorced individuals scored highest in soft skills and thriving at work, followed by widowed individuals, with single participants scoring the lowest. Also, nursing diploma holders scored highest in soft skills and thriving at work, followed by bachelor of nursing holders and nursing technicians (F = 12.483, *p* < 0.001 for soft skills; F = 26.478, *p* < 0.001 for thriving at work). Nurses with less than 1 year of experience scored highest, while those with 1- <5 years of experience scored lowest. Similar trends were observed for experience in the current hospital (F = 26.616, *p* < 0.001 and F = 50.926, *p* < 0.001 for years of experience as a nurse; F = 18.505, *p* < 0.001 and F = 34.275, *p* < 0.001 for years of experience in the current hospital).

**Table 3:** The influence of socio-demographic variables on the OI, soft skills, and thriving at work Scale (*n* = 740)

socio-demographic characteristics	No	%	Soft skill evaluation		Thriving at work	
			Mean ± SD	Test of sig.	Mean ± SD	Test of sig.
**Age (years)**						
< 20	116	15.7	94.96 ± 22.58		85.66 ± 9.45	
20–30	168	22.7	100.92 ± 14.70	F = 25.640*	83.94 ± 7.34	F = 23.006*
> 30–40	209	28.2	100.50 ± 17.44	*p* < 0.00**	84.51 ± 6.57	*p* < 0.00**
> 40–50	122	16.5	101.28 ± 16.70		89.63 ± 10.75	
> 50	125	16.9	115.45 ± 11.36		91.14 ± 6.32	
**Gender**						
Males	227	30.7	96.81 ± 17.97	t = 5.747*	83.09 ± 7.60	t = 7.615*
Females	513	69.3	104.84 ± 17.32	*p* < 0.00**	88.04 ± 8.40	*p* < 0.00**
**Marital status**						
Single	149	20.1	100.87 ± 20.75		85.90 ± 8.36	
Married	470	63.5	101.18 ± 17.77	F = 8.468	86.16 ± 8.65	F = 4.536*
Divorced	60	8.1	105.08 ± 10.95	*p* < 0.00**	87.27 ± 7.68	*p* = 0.004**
Widow	61	8.2	112.66 ± 12.85		90.16 ± 7.33	
**Qualification**						
Nursing diplom	301	40.7	105.29 ± 18.31	F = 12.483*	88.92 ± 9.61	F = 26.478*
Nursing technicians	211	28.5	97.46 ± 17.96	*p* < 0.00**	86.15 ± 7.40	*p* < 0.00* *
Bachelor of nursing	228	30.8	103.09 ± 16.34		83.71 ± 6.75	
**Years of experience as a nurse (years)**						
< 1	21	2.8	116.38 ± 1.75		91.48 ± 2.18	
1- <5	223	30.1	100.84 ± 20.08		84.68 ± 7.61	
5 - <10	165	22.3	93.37 ± 17.92	F = 26.616*	83.50 ± 7.92	F = 50.926*
10 - <15	123	16.6	102.08 ± 8.07	*p* < 0.00**	83.07 ± 6.67	*p* < 0.00* *
≥ 15	208	28.1	109.94 ± 16.53		92.44 ± 7.89	
**Years of experience in this hospital**						
< 1	54	7.3	92.17 ± 24.97		81.59 ± 10.79	
1- <5	273	36.9	101.82 ± 17.86		84.78 ± 6.36	
5 - <10	124	16.8	95.31 ± 15.86	F = 18.505*	86.19 ± 7.78	F = 34.275*
10 - <15	102	13.8	105.20 ± 9.30	*p* < 0.00**	84.17 ± 8.69	*p* < 0.00*
≥ 15	187	25.3	109.29 ± 17.28		92.01 ± 8.19	

Note: Data are *n* (%) and *Mean* ± *SD*. F: One way ANOVA test t: Student t-test *: Statistically significant at *p* ≤ 0.05 **: High Statistically significant at *p* ≤ 0.001

Table 4 shows the correlation between Organizational Intelligence (OI), soft skills, and thriving at work. The correlation between OI and soft skills was strong (*r* = 0.777, *p* < 0.001), indicating a significant positive relationship. Similarly, OI had a positive correlation with thriving at work (*r* = 0.293, *p* < 0.001), and soft skills also correlated positively with thriving at work (*r* = 0.342, *p* < 0.001).

**Table 4:** The correlation between OI, soft skills, and thriving in the workplace. (*n* = 740)

		Organizational intelligence questionnaire	Soft skill evaluation
**Organizational intelligence questionnaire**	r		
	*p*		
**Soft skill evaluation**	r	0.777*	
	*p*	< 0.001*	
**Thriving at work**	r	0.293*	0.342*
	*p*	< 0.001*	< 0.001*

r: Pearson coefficient *: Statistically significant at *p* ≤ 0.05 **: High Statistically significant at *p* ≤ 0.001

Table 5 presented a stepwise regression analysis showing the effect of OI and demographic characteristics on soft skills. The model explained 65.3% of the variance in soft skills (Adjusted R Square = 0.653). Among the demographic characteristics, age, gender, education, experience in the nursing profession, and experience in the hospital position significantly affected soft skills. Specifically, for every one-year increase in age, soft skills increased by 3.824 units (*p* < 0.001). The effect of gender on soft skills was also significant, with a coefficient of 2.658 (*p* = 0.008), suggesting that there may have been differences in soft skills between genders. Education hurt soft skills, with a coefficient of -1.259 (*p* = 0.011), indicating that soft skills decreased as the level of education increased. Experience in the nursing profession had a strong negative effect on soft skills, with a coefficient of -7.531 (*p* < 0.001), suggesting that as experience in the nursing profession increased, soft skills decreased. On the other hand, experience in the hospital position positively affected soft skills, with a coefficient of 4.880 (*p* < 0.001), indicating that as experience in the hospital position increased, soft skills increased. Finally, organizational intelligence had a robust positive effect on soft skills, with a coefficient of 0.398 (*p* < 0.001), suggesting that as organizational intelligence increased, soft skills increased.

**Table 5:** Effect of organizational intelligence and demographic characteristics on soft skills based on stepwise regression

Model		Unstandardized Coefficients		Standardized Coefficients	t	Sig.	95.0% Confidence Interval for B		R	R Square	Adjusted R Square	Std. Error of the Estimate
		B	Std. Error	Beta			Upper Bound	Lower Bound				
1	**(Constant)**	27.435	2.893		9.484	< 0.001*	21.756	33.115	.810^a^	0.657	0.653	10.53
	**age**	3.824	0.748	0.278	5.111	< 0.001*	2.355	5.293				
	**Gender**	2.658	0.998	0.069	2.663	0.008*	0.699	4.617				
	**marital**	− 0.155	0.709	− 0.007	− 0.219	0.827	-1.547	1.236				
	**education**	-1.259	0.492	− 0.059	-2.557	0.011*	-2.225	− 0.292				
	**Experience in nursing profession**	-7.531	1.085	− 0.527	-6.938	< 0.001*	-9.661	-5.400				
	**Experience in hospital position**	4.880	1.027	0.365	4.750	< 0.001	2.863	6.897				
	**Organizational intelligence**	0.398	0.012	0.730	32.517	< 0.001	0.374	0.422				

t: student t test. R^2^: Coefficient of determination. B: Unstandardized Coefficients. Beta: Standardized Coefficients t: t-test of significance

LL: Lower limit UL: Upper Limit. * Correlation is significant at the 0.05 level (2-tailed)

Table 6 presented a stepwise regression analysis examining the effect of organizational intelligence and demographic characteristics on nurses’ thriving. The model explained 65.3% of the variance in nurses’ thriving (Adjusted R Square = 0.653). Among the demographic characteristics, age, gender, education, experience in the nursing profession, and experience in the hospital position significantly affected nurses’ thriving. Specifically, for every one-year increase in age, nurses’ thriving increased by 3.824 units (*p* < 0.001). The effect of gender on nurses’ thriving was also significant, with a coefficient of 2.658 (*p* = 0.008), suggesting that there may have been differences in nurses’ thriving between genders. Education had a negative effect on nurses’ thriving, with a coefficient of -1.259 (*p* = 0.011), indicating that as the level of education increased, nurses’ thriving decreased. Experience in nursing had a strong negative effect on nurses’ thriving, with a coefficient of -7.531 (*p* < 0.001), suggesting that as experience in nursing increased, nurses’ thriving decreased.

**Table 6:** Effect of organizational intelligence and demographic characteristics on nurses’ thriving based on stepwise regression

Model		Unstandardized Coefficients		Standardized Coefficients	t	Sig.	95.0% Confidence Interval for B		R	R Square	Adjusted R Square	Std. Error of the Estimate
		B	Std. Error	Beta			Upper Bound	Lower Bound				
1	**(Constant)**	27.435	2.893		9.484	< 0.001*	21.756	33.115	.810^a^	0.657	0.653	10.53
	**age**	3.824	0.748	0.278	5.111	< 0.001*	2.355	5.293				
	**sex**	2.658	0.998	0.069	2.663	0.008*	0.699	4.617				
	**marital**	− 0.155	0.709	− 0.007	− 0.219	0.827	-1.547	1.236				
	**education**	-1.259	0.492	− 0.059	-2.557	0.011*	-2.225	− 0.292				
	**Experience in nursing profession**	-7.531	1.085	− 0.527	-6.938	< 0.001*	-9.661	-5.400				
	**Experience in hospital position**	4.880	1.027	0.365	4.750	< 0.001*	2.863	6.897				
	**Organizational intelligence**	0.398	0.012	0.730	32.517	< 0.001*	0.374	0.422				

t: student t test. R^2^: Coefficient of determination. B: Unstandardized Coefficients. Beta: Standardized Coefficients t: t-test of significance

LL: Lower limit UL: Upper Limit. * Correlation is significant at the 0.05 level (2-tailed)

## Discussion

Organizational intelligence is regarded as one of the requirements for successful organizations and aids them in fulfilling their goals. Also, it assists in increasing adaptability, collaboration, and diversity [19, 20]. The results of the current study suggest that the nurses studied had a moderate level of organizational intelligence according to their perception. Furthermore, the Culture dimension exhibited the highest mean score. The hospital’s ability to respond to patient’s needs and treat them fairly and equally, its multidisciplinary team, managers’ clear direction, the enhancement of ethics and integrity within the hospital, the trusting environment within the hospital, and the nurses’ adherence to policies and teamwork are all possible contributing factors to these findings.

On the contrary, the lowest mean score is the measures and reward dimension; nurses must be aware of all aspects of performance evaluation and limited promotion and reward, especially for practical nurses. In the same concern, Harhash et al. (2021) found that the mean score of organizational culture is that more than half of the studied nurses had a solid organizational culture [20]. On the other hand, Bilgen et al. (2022) showed low perception and knowledge limitations about organizational intelligence [1].

Moreover, the study revealed that nurses highly perceived soft skills. Additionally, the documentation dimension obtained the highest mean score. This is due to the studied nurses being aware that documentation of everything on time is indispensable and documenting all procedures performed accurately and concisely. According to the systematic review of Jamaludin et al. (2021), nurses need soft skills to enhance their ability to organize and prioritize effective patient care delivery in a demanding healthcare environment [21]. On the contrary, Hussein et al. (2021) showed only 22% of nurses had a perception of soft skills before implementing the training program [18]. In addition, according to Demsash et al. (2023), 32.52% of the inadequate everyday practice documentation by health professionals was due to incomplete papers [22].

Furthermore, the studied nurses had a high perception of thriving at work, meaning they were alert and awake, feeling energetic when performing their tasks, and could continuously learn. Peters et al. (2021) demonstrated that beneficial health and well-being create strong workforces and workplaces where employees thrive both at work and in their lives outside of work [23].

The results of this study specified that divorced nurses scored highest in soft skills and thriving at work, followed by widowed individuals, with single participants scoring the lowest. This may be due to the postulation that individuals who have experienced divorce or the loss of a spouse may undergo significant personal growth and resilience-building processes. These experiences can lead to increased adaptability, emotional intelligence, and the development of coping mechanisms, which positively impact their soft skills and ability to thrive in the workplace. Also, divorced and widowed individuals may have access to support networks that provide emotional support and assistance. These networks can foster a sense of belonging and provide resources that contribute to their well-being and professional development.

Another possible explanation is that Life-altering events like divorce or the loss of a spouse can shift an individual’s perspective and priorities. They may become highly motivated to rebuild their lives and achieve personal and professional success. This increased motivation and goal orientation can positively impact their performance and thriving in the workplace. The experiences and responsibilities of being divorced or widowed may provide individuals with unique perspectives and skills. These experiences contribute to enhanced empathy, communication abilities, problem-solving skills, and a greater appreciation for work-life balance, all critical components of soft skills and thriving at work. This result contradicts Hussein et al. (2021), who found that there were satisfactory soft skills among the majority of married studied nurses [18].

The results of this study indicate a robust statistical relevance between nurses’ perception of all measures of organizational intelligence and the underlying dimensions of soft skills and thriving at work. These findings can be attributed to various factors, including effective communication among nurses, autonomy in performing job duties, involvement in decision-making processes, access to necessary information, clear communication of goals and objectives by top management, respect for every person on the healthcare team, ethical and polite behavior towards patients, and top management’s ability to inspire individuals to implement the hospital’s strategy. On the same line, Rezaei and Mohammadinia (2022) revealed a statistically significant difference between OI and how to communicate with patients [24]. Furthermore, Sokhtsaraei (2019) shows a significant relationship between organizational intelligence and organizational health and the performance and skills of employees in health [25]. Moreover, Mortezaei et al. (2022) revealed a positive and significant relationship between intelligence and hospital innovation. In other words, using intelligence in a hospital will lead to more organizational innovation [26].

Additionally, Organizational intelligence, soft skills, and thriving at work are positively correlated across all sociodemographic categories with a statistically significant. Furthermore, an analysis revealed that nurses with a bachelor’s degree in nursing and more than 15 years of experience achieved a notably elevated organizational intelligence score. This may be attributed to providing support and opportunity for advancement and development, a heightened power level, and the capacity to make precise and informed decisions.

On the other hand, according to stepwise regression, It was an astonishing result as nurses’ education and experience hurt their soft skills and thriving, indicating that as the level of education and experience increased, soft skills and thriving decreased.

The researchers expected nurses’ education and experience to positively affect their overall skills and professional development. However, this may be rationalized by the assumption that nursing education often focuses on developing technical skills and clinical knowledge. While these are crucial for providing quality patient care, the emphasis on technical aspects may need to be revised to include developing soft skills such as communication, empathy, and teamwork. As a result, nurses may need more training or support to enhance their soft skills, essential for building strong relationships with patients and colleagues. More to the same point, nurses who have been in the profession for a long time may need more opportunities for career advancement or continuing education. This lack of professional growth can contribute to feelings of stagnation and dissatisfaction, affecting their m, motivation and engagement at work. With avenues for ongoing learning and development, nurses can adapt to changing healthcare practices and may have the opportunity to enhance their soft skills. These findings are consistent with those of Bratajaya (2021), who found that senior nurses are more aware of nine crucial soft skills—self-control, self-motivation, adaptability, flexibility, hospitality, and presentation skills than beginner nurses. Additionally, they highlight the significance of these soft skills and how they function as a crucial intermediate stage between beginner and senior nursing [12].

The study’s result showed that nurses with more than 15 years of hospital experience had a notably elevated level of proficiency in soft skills and flourished in the nursing field. This can be rationalized by the fact that nursing is a demanding profession, with long working hours, high patient loads, and exposure to emotionally challenging situations. Experienced nurses may have accumulated years of exposure to deal with similar situations, which can lead to easier decisions and affect their ability to thrive in the workplace. High levels of stress can also impact interpersonal soft skills.

### Strengths and limitations

There are approximately returns to this study. By assessing several variables in the population sample at one time, the cross-sectional design made it possible to get precise data that was less susceptible to the possible prejudices in case reports and case series. The study adds to the understanding of organizational intelligence, soft skills, and thriving as experienced by nurses, an area of understudied interest in the healthcare industry.

There are, nevertheless, certain restrictions. Firstly, because the study was conducted at a single hospital, generalization is not possible. Second, the association between nurses’ perceptions of organizational organizational intelligence and soft skills and their views of thriving at work as a dependent variable was the only one examined in this study. Future studies can assess additional variables that impact the thriving of nurses’ work. Furthermore, the questionnaire’s paper-based data entry and cleaning were highly labor-intensive. Lastly, there is no suggestion of causation between the elements in the study. Well, it is created to investigate the connection between variables.

## Conclusion

The findings underscore the significance of organizational support and leadership in fostering a positive work environment that promotes the development of organizational intelligence and learning soft skills among nurses. Nursing is a career that stands out for its compassionate amenities and human-centeredness. Only nurses interact with patients and their families by specific values [27]. This suggests that healthcare organizations should prioritize creating a supportive culture that values continuous learning and skill development. Hospital leaders should inspire nursing leaders and subordinates’ organized communication networks, stirring nurses’ involvement in committee gatherings and decision-making [28]. Overall, this cross-sectional study expands our understanding of the relationships between organizational intelligence, soft skills, and thriving in nursing, highlighting their interconnectedness and emphasizing the importance of these factors in promoting nurses’ well-being and success.

The total score for organizational intelligence was moderate, and nurses have a modest level of soft skills and thrive at work, as they perceived. In addition, the results concluded a highly significant strong positive association between the scores of organizational intelligence and the soft skills scale. Furthermore, a noteworthy positive connection was seen between the score of organizational intelligence and thriving in work.

### Implications in nursing practice

- This cross-sectional study on the nexus between organizational intelligence, soft skills, and thriving in nursing has shed light on several significant findings. First, organizational intelligence plays a crucial role in enhancing nurses’ overall performance and well-being. Second, soft skills, such as effective communication and emotional intelligence, are essential for nurses to thrive. This finding has implications for healthcare organizations and policymakers, who should prioritize developing and cultivating organizational intelligence and soft skills among nurses.
- In addition, the study emphasizes the need for comprehensive training programs and interventions to enhance nurses’ soft skills and organizational intelligence. By providing nurses with the necessary knowledge, skills, and resources, healthcare organizations can create an environment that supports their growth and flourishing. This can improve patient care, reduce burnout, and increase nurse job engagement.
- Furthermore, the study underscores the importance of considering individual and organizational factors to promote nurses’ thriving. Combining personal attributes (soft skills) and the organization’s characteristics (organizational intelligence) contribute to nurses’ well-being and professional success. Healthcare leaders should recognize the interplay between these factors and develop strategies addressing individual and organizational aspects to foster nurses’ thriving.

## Data Availability

The datasets generated during and analyzed during the current study are available from the corresponding author upon reasonable request.
